# Occurrence of mycotoxins in feed ingredients and complete feeds obtained from the Beijing region of China

**DOI:** 10.1186/2049-1891-5-37

**Published:** 2014-07-22

**Authors:** Xiaoying Li, Lihong Zhao, Yu Fan, Yaxiong Jia, Lei Sun, Shanshan Ma, Cheng Ji, Qiugang Ma, Jianyun Zhang

**Affiliations:** 1 State Key Laboratory of Animal Nutrition, China Agricultural University, No. 2. West Road Yuanming Yuan, Beijing 100193, P.R. China; 2 Beijing Municipal General Station for Animal Husbandry and Veterinary Service, Beijing, China; 3 Institute of Plant Protection, Chinese Academy of Agricultural Sciences, Beijing, China

**Keywords:** AflatoxinB_1_, Beijing region, Deoxynivalenol, Ochratoxin A, Swine feeds, Zearalenone

## Abstract

**Background:**

The current study was carried out to provide a reference for the control of mycotoxin contamination in feed ingredients and complete feeds for swine.

**Methods:**

A total of 55 feed ingredients, including 14 corn, 13 wheat bran, 11 soybean meal and 17 dried distillers grains with solubles (DDGS) as well as 76 complete swine feeds including 7 creep feeds, 14 starter feeds, 14 grower feeds, 18 grower-finisher feeds, 10 gestating sow feeds, and 13 lactating sow feeds were randomly collected from 15 swine farms located in the Beijing region of China from July to August 2011. Immunoaffinity clean-up, using High-Performance Liquid Chromatography (HPLC) in combination with UV or Fluorescence Detection, was used for quantitative analysis of aflatoxin B_1_ (AFB_1_), deoxynivalenol (DON), zearalenone (ZEA) and ochratoxin A (OTA) in the ingredients and complete feeds.

**Results:**

DON and ZEA were the most prevalent mycotoxins found. DON was detected at percentages of 93, 92, 54, 100 and 97% with a mean level of 1.01, 0.44, 0.05, 1.36 and 0.65 ppm in the samples of corn, wheat bran, soybean meal, DDGS and complete feeds, respectively. The detected percentages of ZEA were 100, 100, 54, 100 and 100 with mean levels of 109.1, 14.9, 9.2, 882.7 and 58.9 ppb in the same samples. In the wheat bran and soybean meal samples, the content of all four mycotoxins were below the maximum limits set by Chinese regulations while the percentage of samples that exceeded regulatory limits were 7, 57 and 7% for corn, and 7, 14 and 3% for the complete feeds for AFB_1_, DON and OTA respectively. DDGS showed the most serious mycotoxin contamination and the percentage of samples that exceeded regulatory limits were 6, 88 and 41%, for AFB_1_, DON and ZEA, respectively.

**Conclusions:**

This paper is the first to present data on the natural occurrence of AFB_1_, DON, ZEA and OTA in ingredients and complete feeds obtained from swine farms in China’s Beijing region. The data shows that feed ingredients and complete swine feeds obtained from these farms are most often contaminated with DON followed by contamination with AFB_1_ and ZEA.

## Background

Mycotoxins are a large group of fungal metabolites found worldwide in cereal grains and other feed products. Mycotoxins pose a severe threat to humans and as well as causing huge economic losses in the feed and food industries [1]. Aflatoxin, deoxynivalenol (DON), zearalenone (ZEA) and ochratoxin A (OTA) are considered the most economically important mycotoxins in terms of their prevalence and their negative effects on animal performance [2,3]. Pigs are particularly susceptible to mycotoxins, suffering a variety of chronic or acute syndromes depending on the amount of contaminants they consume [4].

Aflatoxins are the most potent of the known mutagenic and carcinogenic natural substances produced by *Aspergillus* spp. [5]. There are six types of aflatoxins that frequently contaminate feeds and foods, including B_1_, B_2_, G_1_ and G_2_. Aflatoxin B_1_ (AFB_1_) has long been considered one of the most poisonous carcinogens [6]. A potent hepatocarcinogenic and hepatotoxic mycotoxin [7], it has been placed in Group ΙA by the International Agency for Research on Cancer IARC: [8].

DON is one of the most frequently detected trichothecene contaminants in cereals [9]. The ingestion of feeds contaminated with DON leads to feed refusal and weight loss, decreases nutritional efficiency and causes lesions in the gastrointestinal tract, vomiting, bloody diarrhea and severe dermatitis accompanied by hemorrhaging and immune dysregulation. Swine are typically the most sensitive of the susceptible species in that they exhibit the most severe symptoms of acute DON toxicity [10,11].

ZEA is a non-steroidal estrogenic toxin produced by certain *Fusarium* species [12]. ZEA can competitively bind to estrogen receptors leading to reproductive disorders and estrogenic dysfunction in humans and animals (especially in breeding animals), impairing fertility and increasing the frequency of stillbirths along with reducing sperm quality [12,13]. Swine are the animal species most severely affected by ZEA [14,15].

OTA, produced by the *Penicillium verrucosum* fungus and various species of *Aspergillus*[5] is a nephrotoxic mycotoxin that causes renal toxicity and possesses carcinogenic, teratogenic immunotoxic and possibly neurotoxic properties [6]. This toxin has been classified by the IARC as a possible human carcinogen [8]. Research on the effects of this mycotoxin in swine has revealed that it causes changes in renal function [16].

China faces a feed shortage. The feed that is available is frequently contaminated with mycotoxins [17]. Furthermore, the high prices and scarcity of protein sources for animal feeds have led to the use of alternative protein sources such as distillers dried grains with solubles (DDGS). However, the supplementation of animal diets with DDGS must be carefully monitored for the presence of toxic compounds such as mycotoxins, especially AFB_1_, DON, ZEA and OTA [18].

Although Beijing is one of the largest cities in China, there has been little research conducted on the presence of the mycotoxins AFB_1_, DON, ZEA and OTA in the Beijing region. As the surveys that have been done have been limited and because more thorough research is very much necessary, the current study investigated the occurrence of four major mycotoxins in feed ingredients (corn, wheat bran, soybean meal and DDGS) and complete swine feeds for various stages of growth on a variety of swine farms in China’s Beijing region.

## Methods

### Sample collection and preparation

The protocol for the experiment was reviewed and approved by the Institutional Animal Care and Use Committee at China Agricultural University.

A total of 131 samples including 55 feed ingredients and 76 complete swine feeds were randomly sampled from 15 swine farms in the Beijing region from July through August 2011. The samples included 16 samples of corn, 13 samples of wheat bran, 12 samples of soybean meal, and 17 DDGS samples. The swine complete feeds included creep feeds (n = 7), starter feeds (n = 14), grower feeds (n = 14), grower-finisher feeds (n = 18), gestating sow feeds (n = 10) and lactating sow feeds (n = 13). The sampling was undertaken in accordance with European Regulation No. 401/2006 [19]. All samples were ground, mixed and stored at 4°C prior to analysis.

### Methods of analysis for mycotoxins

#### AFB_1_ analysis

Samples were analyzed according to the methods of the AOAC [20]. Briefly, 25 g of ground feed was extracted with 100 mL of methanol:water (80:20, v/v), blended at high speed for 3 min and then filtered through a sheet of Waterman Filter Paper No. 4. The extract was diluted with a phosphate-buffered saline solution (PBS, pH 7.40), mixed well and filtered through microfiber filter paper. The immunoaffinity column (AokinImmunoClean CF AFLA, Aokin AG, Berlin, Germany) was conditioned with 1 mL of sodium azide, and 10 mL of the diluted filtrate was passed through the column at a flow rate of 1 mL/min. The column was then washed with 10 mL of a methanol:water solution (10:90, v/v) at a flow rate of 3 mL/min. The retained chemicals were then eluted with 1 mL of methanol at a flow rate of 1 mL/min. Subsequently, 20 μL of the clear eluate was injected directly into an HPLC system. If the eluate was found not to be clear, it was passed through an organic filter unit (0.45 μm) before injection. The mobile phase utilized a methanol:water solution (50:50, v/v) with the flow rate set at 1 mL/min. Post-column derivatization was performed with a photochemical reactor (AURA, Los Angeles, CA). A C18 column (4.6 mm × 150 mm, 5 μm, Dikma, Beijing, China) was employed with the LOD set at 0.03 ppb and the LOQ at 0.1 ppb. A fluorescence detector (SHIMADZU, Kyoto, Japan) was set for excitation and emission wavelengths of 360 and 440 nm, respectively. The retention time was 16.5 min.

#### DON analysis

Samples were analyzed according to the method of GB/T 23503–2009. Briefly, 25 g of ground feed was extracted with 100 mL of a solution of methanol:water (60:40, v/v), blended at high speed for 3 min and then filtered through a sheet of Waterman Filter Paper No. 4. The extract was diluted with a phosphate-buffered saline solution (PBS, pH 7.4), mixed well and filtered through microfiber filter paper. The immunoaffinity column (AokinImmunoClean CF DON, Aokin AG, Berlin. Germany) was conditioned with 1 mL of sodium azide, and 10 mL of the diluted filtrate was passed through the column by gravity at a flow rate of 1 mL/min. The column was then washed with a 10 mL solution of methanol:water (10:90, v/v) at a flow rate of 3 mL/min. The bound compounds were then eluted with 3 mL of methanol at a flow rate of 1 mL/min. The purified samples were dried under a stream of nitrogen gas at 50°C. For the mobile phase, the residue was dissolved in 200 μL after evaporation. Subsequently, 20 μL of the eluate was injected into an HPLC system. The mobile phase utilized a methanol: water solution (30: 70, v/v)with the flow rate set at 1 mL/min. A C18 column (4.6 mm × 150 mm, 5 μm, Agilent, Santa Clara, CA) was employed with the LOD set at 0.02 ppm and the LOQ at 0.06 ppm. The absorption UV wavelength (SHIMADZU, Kyoto, Japan) was set at 218 nm. The retention time was 5.6 min.

#### ZEA analysis

Samples were analyzed according to the certified Chinese GB/T 23504–2009 method. Briefly, 25 g of feed was extracted with 100 mL of a methanol:water solution (60:40, v/v), blended at high speed for 3 min and then filtered through a sheet of Waterman Filter Paper No. 4. The extract was diluted with a phosphate-buffered saline solution (PBS, pH 7.40), mixed well and filtered through microfiber filter paper. The immunoaffinity column (AokinImmunoClean CF ZEA, Aokin AG, Berlin, Germany) was conditioned with 1 mL of sodium azide, and 10 mL of the diluted filtrate were passed through the column by gravity at a flow rate of 1 mL/min. The column was then washed with 10 mL of a methanol:water solution (10:90, v/v) at a flow rate of 3 mL/min. The bound chemicals were then eluted with 3 mL of methanol at a flow rate of 1 mL/min. Subsequently, 20 μL of the clear eluate was injected directly into an HPLC system. If the eluate was found not to be clear, it was passed through an organic filter unit (0.45 μm) before injection. The mobile phase utilized an acetonitrile:water:methanol solution (46: 46: 8, v/v/v with the flow rate set at 1 mL/min. A C18 column (4.6 mm × 150 mm, 5 μm, Dikma, Beijing, China) was employed with the LOD set at 1.5 ppb and the LOQ at 4 ppb. A fluorescence detector (SHIMADZU, Kyoto, Japan) was set for excitation and emission wavelengths of 274 and 440 nm, respectively. The retention time was 7.3 min.

#### OTA analysis

Samples were analyzed according to the methods of the AOAC [21]. Briefly, 25 g of feed was extracted with 100 mL of a methanol:water solution (60:40, v/v), blended at high speed for 3 min and then filtered through a sheet of Waterman Filter Paper No. 4. The extract was diluted with a phosphate-buffered saline solution (PBS, pH 7.4), mixed well and filtered through microfiber filter paper. The immunoaffinity column (AokinImmunoClean CF OTA, Aokin AG, Berlin, Germany) was conditioned with 1 mL of sodium azide, and 10 mL of the diluted filtrate was passed through the column by gravity at a flow rate of 1 mL/min. The column was then washed with 10 mL of a methanol:water solution (10:90, v/v) at a flow rate of 3 mL/min. The bound compounds were then eluted with 3 mL of methanol at a flow rate of 1 mL/min, after which 20 μL of the clear eluate was injected directly into an HPLC system. If the eluate was found not to be clear, it was passed through an organic filter unit (0.45 μm) before injection. The mobile phase utilized an acetonitrile:water:glacial acetic acid solution (99: 99: 2, v/v/v) with the flow rate set at 0.9 mL/min. A C18 column (4.6 mm × 150 mm, 5 μm, Dikma, Beijing, China) was employed with the LOD set at 0.035 ppb and the LOQ at 0.117 ppb. A fluorescence detector was set for excitation and emission wavelengths of 333 and 477 nm, respectively. The retention time was 11.4 min.

### Validation of analytical methods

The analytical methods used were evaluated for linearity, recovery and reproducibility. Standard curves were generated by comparing the linear regressions of peak regions against concentrations. The recovery (precision) of mycotoxins was determined by spiking blank samples (corn, wheat bran, soybean meal, DDGS and complete feeds) with known levels of AFB_1_, DON, ZEA or OTA standards dissolved during the mobile phase. The spiked concentrations of AFB_1_, DON, ZEA and OTA were evaluated at high, intermediate and low levels for each standard mycotoxin concentration. Each sample was analyzed after being allowed to stand for 2 h to allow solvent equilibration between the analytes and the matrix. The reproducibility of the method was established by injecting five replicates of the same standard solution on the same day and over five different days.

### Statistical analysis

The data were analyzed using MS Excel, expressed as average contents, detection rates and the percentage of samples that exceeded regulatory limits.

## Results and discussion

### Performance of the analytical methods

The standard curves for AFB_1_, DON, ZEN and OTA produced from three determinations of five concentration levels were linear with correlation coefficients (R^2^) ranging from 0.9990 to 0.9999. Both the intra-day and inter-day precision measurements showed relative standard deviations below 15%. The determination of recoveries in the blank samples spiked with AFB_1_, DON, ZEN and OTA were 80.3 to 85.0, 84.1 to 89.8, 86.6 to 90.4 and 87.3 to 90.8, respectively. Therefore, the performances of the employed analytical methods were shown to be satisfactory for the study’s purposes. The analytical methods were validated for all feed samples and met the performance criteria set by European Regulation No. 401/2006 [14] for mycotoxin analysis.

### The occurrence of mycotoxins in feed ingredients and complete feeds

#### Corn

The occurrence of mycotoxin contamination in corn is summarized in Table 1. AFB_1_ was detected in 50% of the corn samples and 7% of the corn samples showed concentrations exceeding regulatory limits. The highest concentration of AFB_1_ in corn was 58.9 ppb while the average value was 6.0 ppb. The corn samples were also contaminated with DON (93% detection rate). The percentage of corn samples exceeding regulatory limits for DON was very high, with 57% of samples exceeding the maximum limits set by Chinese regulations. The average content of ZEA in corn was 109.1 ppb and the average value of OTA was 22.1 ppb. Additionally, the percentage of corn samples exceeding regulatory limits for OTA was 7%.

**Table 1:** **Occurrence of mycotoxins in feed ingredients and complete swine feeds**^**1**,**2**^

**Item**	**Total samples**, **n**	**Average contents**				**Content range**				**Detection rates, %**				**Percentage of samples over the maximum limits, %**^**1**,**2**^			
		**AFB**, **ppb**	**DON**, **ppm**	**ZEA**, **ppb**	**OTA**, **ppb**	**AFB**_**1**_, **ppb**	**DON**, **ppm**	**ZEA**,**ppb**	**OTA**,**ppb**	**AFB**_**1**_	**DON**	**ZEA**	**OTA**	**AFB**_**1**_	**DON**	**ZEA**	**OTA**
Corn	14	5.98	1.01	109	22	<LOQ-58.9	<LOQ-2.13	6.25-321	<LOQ-136	50	93	100	93	7	57	0	7
Bran	13	0.25	0.44	159	8	<LOQ-1.1	<LOQ-0.89	<LOQ-44.4	<LOQ-36	46	92	100	77	0	0	0	0
Soybean meal	11	1	0.05	9	11	<LOQ-10.7	<LOQ-0.21	<LOQ-35.4	<LOQ-42	36	54	54	64	0	0	0	0
DDGS	17	9.83	1.36	883	23	<LOQ-64.1	0.85-1.72	48.89-2958	1.79-89	94	100	100	100	6	88	41	0
Creep feed	7	0.54	0.28	39	5	<LOQ-3.7	<LOQ-0.53	4.7-107	<LOQ -22	57	86	100	100	0	0	0	0
Starter feed	14	0.51	0.85	54	16	0.1-3.9	0.16-2.31	<LOQ-115	<LOQ-84	71	100	100	86	0	14	0	0
Grower feed	14	0.31	0.62	67	12	<LOQ-3.9	0.12-1.14	7.59-132	<LOQ-94	57	100	100	86	0	14	0	0
Grower-finisher feed	18	4.33	0.58	59	13	<LOQ-38.3	<LOQ-1.44	7.1-150	<LOQ-92	67	94	100	94	11	22	0	0
Gestating sow feed	10	0.25	0.82	63	48	<LOQ-1.64	0.13-1.45	9.2-149	<LOQ-212	70	100	100	90	0	10	0	20
Lactation sow feed	13	9.55	0.62	76	15	<LOQ-41.0	0.07-1.52	7.4-231	1.67-92	69	100	100	100	23	23	0	0
Total	76	2.89	0.65	59	18	<LOQ-41.0	<LOQ-2.31	<LOQ-232	<LOQ-212	70	97	100	92	7	14	0	3

^1^ Samples exceeding regulatory limits of four mycotoxins: higher than the maximum limits set by the Chinese regulation (Table 2).

^2^ Maximum limit of mycotoxins in other feed ingredients is in accordance with the regulations on mycotoxins in corn.

Corn is an energy ingredient frequently used in animal feeds in China. The results showed that corn is frequently contaminated with AFB_1_, DON, ZEA and OTA. The percentages of samples in which AFB_1_ exceeded regulatory limits were similar to those in corn samples surveyed by Rodrigues and Naehrer in North, South-East and South Asia and in Oceania [22]. The results also showed that corn samples were contaminated with DON at an average value of 1.01 ppm while the highest value detected was 2.13 ppm which agrees with the maximum value in corn reported by Rodriguez and Naehrer [22]. The average content of ZEA in corn was similar to the 121 ppb reported by the two researchers in China [22]. It was similar to the concentrations of DON (93.20%) and ZEA (96.60%) in corn as reported by Guan et al. [17]. The average values and concentrations of OTA in the samples that exceeded regulatory limits was in agreement with survey results that have shown lower contamination levels of OTA than other mycotoxins in corn [5,23].

#### DDGS

The occurrence of mycotoxin contamination with DDGS is summarized in Table 1. The results showed that 6% of the DDGS contained concentrations of AFB_1_ that exceeded regulatory limits, with an average content of 9.8 ppb. Approximately 88% of the samples contained concentrations of DON that exceeded regulatory limits with an average content of 1.4 ppm. The ZEA contamination levels in the DDGS samples ranged from 49 to 2,958 ppb. We found that 41% of the samples contained concentrations of ZEA that exceeded regulatory limits, with an average content of 883 ppb. However, the OTA content in the DDGS samples did not exceed the standard limits. These samples had an average OTA content of 23 ppb.

The occurrence of mycotoxins in DDGS used as an ingredient in pig diets has rarely been studied. Four mycotoxins were found to be prevalent in the DDGS samples used in our study. Contamination with DON and ZEN was particularly serious. The percentage of samples containing concentrations of DON that exceeded regulatory limits and the average content of DON detected in these samples were similar to the concentrations of DON that have been found in distillers dried grains with solubles sourced worldwide [18]. These results may be explained by the fact that mycotoxins in DDGS (constituting the remaining portions within the final by-product) are up to three times more concentrated than in corn grain [24]. Furthermore, if improperly stored, DDGS are easily contaminated with more mycotoxins due to their high moisture content. In addition, grains are damaged during the process of DDGS production, which can easily cause the production of more mycotoxins. Moreover, in our study, the DDGS were obtained from the regions lying along the middle and lower reaches of the Yangtze River (in Henan, Sichuan and Anhui provinces) which are characterized by a moist climate which may contribute to the contamination of corn with mycotoxins [25,26]. What is more, this study has shown that multiple mycotoxins coexist in most feeds and feedstuffs [27,28]. About 29% (5/17) of the DDGS samples used in this study were co-contaminated with DON and ZEN at levels of 1 ppm and 500 ppb, respectively, which exceeds Chinese regulatory limits for feedstuffs. The simultaneous occurrence of contamination by various kinds of mycotoxins leads not only to immune suppression in animals, but also lowered efficiency in animal production [29].

#### Wheat bran and soybean meal

The occurrence of mycotoxin contamination in wheat bran and soybean meal is summarized in Table 1. The detection rate of AFB_1_, DON, ZEA and OTA was 46, 92, 100 and 77% in wheat bran, and 36, 54, 54 and 64% in soybean meal, respectively.

The samples of wheat bran and soybean meal were analyzed for the presence of four mycotoxins. The contamination levels of these mycotoxins were lower than those found in the corn and DDGS samples and did not exceed the maximum limits set in China (Table 2). In general, feed ingredients such as soybean meal and wheat bran were less contaminated with mycotoxins, a finding in agreement with the results of a survey on the worldwide occurrence of mycotoxins in feedstuffs and feed conducted by Rodrigues and Naehrer [22]. This may be due to the low moisture content and the composition (high protein/carbohydrate ratio) of soybean meal, which inhibits the growth of mold. Another reason is that producers and farmers pay more attention to storage conditions because of the high price of soybean meal.

**Table 2:** **Maximum limit regulations for mycotoxins in feedstuffs in China**^**1**,**2**^

**Mycotoxin**	**Feedstuff**	**Maximum limit**, **ppb**	**Reference**
AFB_1_	Corn	50	GB1 13078-2001
	Soybean meal	30	GB 13078-2001
	Starter pig feed	10	GB 13078-2001
	Grower pig feed	20	GB 13078-2001
	DDGS	50	NY/T2 1968-2010
DON	Swine complete feed	1,000	GB 13078.3-2007
	Corn	1,000	GB 2761–2011.
	DDGS	1,000	NY/T 1968-2010
ZEA	Corn	500	GB 13078.2-2006
	Complete feed	500	GB 13078.2-2006
OTA	Corn	100	GB 13078.2-2006
	Swine complete feed	100	GB 13078.2-2006
	DDGS	100	NY/T 1968-2010

^1^ *GB*: General Administration of Quality Supervision, Inspection and Quarantine of the People’s Republic of China. National Standard of the People’s Republic of China.

^2^ NY/T: Ministry of Agriculture of the People’s Republic of China. Agricultural Standard of the People’s Republic of China.

#### Complete feeds

The occurrence of mycotoxin contamination in complete swine feed is summarized in Table 1. The incidences of the four investigated mycotoxins ranged from 57 to 100% in complete feeds obtained from swine farms. The detection rate of AFB_1_ in the samples of creep feed, starter pig feed, grower pig feed, grower-finisher pig feed, gestating sow feed and lactating sow feed were 57, 71, 57, 67, 70 and 69%, respectively, with mean concentrations of 0.54, 0.51, 0.31, 4.33, 0.25 and 9.55 ppb, respectively. In addition, the percentage of grower-finisher pig feed and lactating sow feed that contained concentrations of AFB_1_ that exceeded regulatory limits was 11 and 23%, respectively.

The detection rate of DON was also very high, with percentages in creep feed, starter pig feed, grower pig feed, grower-finisher pig feed, gestating sow feed and lactating sow feed of 86, 100, 100, 100, 94, 100 and 100%, with average concentrations of 0.28, 0.85, 0.62, 0.58, 0.82 and 0.62 ppm, respectively. The percentage of samples with DON concentrations exceeding regulatory limits in complete feeds for the various stages of pig growth was 0, 14, 14, 22, 10 and 23%, respectively. The highest concentration of DON was detected in starter pig feeds (2.31 ppm). Moreover, although high incidences of ZEA and OTA (86 to 100%) were found in swine feeds, the mean concentrations were low (that is, they did not exceed the maximum limits set in China), except for the concentrations of OTA found in gestating sow feed.

Although the levels of ZEA and OTA detected in our study did not seem to have any clear clinical effects, there may well be chronic accumulated carry-over effects on animal products over a long period of feed consumption [2]. In contrast, AFB_1_ and DON were found in a high percentage of samples in concentrations that exceeded regulatory limits for swine feeds, which could have clinical effects on gilt and sow health. The fact that AFB_1_ was found at concentrations exceeding regulations in feeds for lactating sows could be of importance in terms of animal’s health. Indeed, it could lead to the excretion of AFM_1_ and to an exposure of piglets to that toxin, since young animals are more sensitive to AFB_1_ than adults. Concentrations as low as 1 ppm may cause loss of appetite, vomiting, low feed efficiency and a reduction in body weight gain [23,30]. Therefore, the high incidence of mycotoxins found in complete feed samples obtained from swine farms highlights the need for surveillance and prevention.

The natural occurrence of mycotoxins has been reported in a variety of foods and feeds in many countries [31]. The maximum admissible levels of these four mycotoxins in feed ingredients and swine complete feeds vary from one country to another with legislation prescribing mandatory upper limits that often take the form of product standards [32]. The United States’ Food and Drug Administration has set a maximum permissible level of 20 ppb for total aflatoxins in all foodstuffs, while in China, the legal limit for AFB_1_ contamination in corn is 50 ppb. The wide variations in standards have resulted in conflicting perceptions regarding safe levels among the various national agencies. The regulatory limits of the four mycotoxins in this study is higher than the maximum limits set by the Chinese regulation (Table 2).

Pigs are among the most sensitive species to all of these toxins [4]. This is also one of the most frequently exposed species due to its diet based mainly on cereals. The regulation is presented as a consequence of pigs sensitivity (in EU, limits for pigs feeds are often lower than those for other species).

The toxicology of mycotoxins and the incidence of mycotoxin contamination have aroused public awareness. Health authorities need to take action to mitigate the serious negative effects they can have on humans and animals in China. The reduction of mycotoxin contamination will require an integrated understanding of agronomy, fungal ecology, harvesting methods, storage conditions, feed processing and detoxification strategies [33,34]. Effective control strategies have been implemented based on the establishment of the Hazard Analysis and Critical Control Point (HACCP). This approach can minimize mycotoxin contamination in the food chain through appropriate management of products [35].

Because mycotoxins are stable chemicals, physical and chemical degradation methods are limited in their effectiveness and include disadvantages such as loss of product nutrition, organoleptic qualities, undesirable health effects and high equipment cost [36]. These disadvantages have encouraged the development of promising biodegradation methods by which mycotoxins may be detoxified [37,38]. Further periodic investigations of feed ingredients and complete feed samples from various regions in China taken during all seasons are ongoing.

This paper is the first to present data on the natural occurrence of AFB_1_, DON, ZEA and OTA in feed ingredients and swine complete feeds obtained from livestock farms in China’s Beijing region. The results of this study should serve as a significant reference for feed manufacturers, animal farms and Chinese regulatory authorities for the future consideration of feed and food safety issues. Periodic surveillance and monitoring of the occurrence of mycotoxins in feed ingredients and in animals are very important for minimizing animal and human health risks.

## Conclusion

The content of all four mycotoxins were below the maximum limits set by Chinese regulations in the wheat bran and soybean meal samples while the percentage of samples that exceeded regulatory limits were 7, 57 and 7% for corn, and 7, 14 and 3% for the complete feeds for AFB_1_, DON and OTA respectively. DDGS exhibited the most serious mycotoxin contamination and the percentage of samples that exceeded regulatory limits were 6, 88 and 41%, for AFB_1_, DON and ZEA, respectively.

## Competing interests

The authors declare that they have no competing interests.

## Authors’ contributions

XYL carried out the statistical analysis and drafted the manuscript. LHZ participated in the chemical analysis. YF, YXJ, LS, SSM, CJ, QGM and JYZ conceived the study, participated in its design and coordination, and helped draft the manuscript. All authors read and approved the final manuscript.
